# Prevalence and predictors of quiet quitting among healthcare professionals: a scale development and validation study

**DOI:** 10.1186/s40359-026-04209-x

**Published:** 2026-02-26

**Authors:** Yasemin Boy, Mahmut Sürmeli, Funda Çam

**Affiliations:** 1 Department of Nursing, Faculty of Health Sciences, Tokat Gaziosmanpasa University, Tokat, Türkiye; 2 Department of Physiotherapy and Rehabilitation, Faculty of Health Sciences, Tokat Gaziosmanpasa University, Tokat, Türkiye; 3 Department of Nursing, Faculty of Health Sciences, Bezmialem Vakif University, İstanbul, Türkiye

**Keywords:** Burnout differentiation, Employee disengagement, Healthcare professionals, Psychometric validation, Quiet quitting syndrome, Work engagement

## Abstract

**Background:**

In recent years, “quiet quitting” (QQ) has emerged as a significant concern in the healthcare sector, reflecting a deeper disengagement from professional roles. Characterized by emotional exhaustion and detachment, QQ is influenced by various demographic and occupational factors. Understanding this phenomenon and its underlying causes is essential for promoting workforce sustainability and well-being. This study aimed to develop a valid and reliable instrument to assess QQ and its determinants among healthcare professionals (HPs).

**Methods:**

A comprehensive literature review was conducted to identify existing research on QQ, and a 25-item draft scale was developed. Following expert evaluation, the scale was administered to 242 HPs for preliminary validation. Exploratory factor analysis and reliability assessments were performed to refine the instrument.

**Results:**

The analysis yielded a refined 20-item scale comprising two subscales: “employee disengagement” and “emotional exhaustion and depersonalization.” The final version was administered to a larger sample of 436 HPs to evaluate the prevalence and causes of QQ. Findings revealed that 29.4% of participants experienced severe QQ. The condition was significantly associated with age, gender, education level, profession, work experience, and parental status. Contributing factors included increased workload (93.8%), unfavorable working conditions (90.6%), psychological violence from managers (90.6%), and low wages (90.6%).

**Conclusions:**

QQ emerges as a multidimensional syndrome encompassing dissatisfaction, emotional exhaustion, and detachment from professional responsibilities. The Quiet Quitting Scale for Health Professionals (QQS-HP) demonstrates strong psychometric properties and provides a valid tool for assessing disengagement in healthcare settings. Early identification and targeted interventions addressing systemic and managerial stressors are crucial for mitigating QQ and enhancing workforce well-being.

## Background

Quiet quitting (QQ) denotes a behavioral and psychological state in which employees disengage from their occupational duties and organizational commitment without overtly expressing discontent or intention to resign [1]. Rather than an outright departure from employment, QQ signifies a subtle yet deliberate reduction in work effort—employees perform only what is strictly required, refraining from extra-role behaviors or organizational participation [2]. The popularization of this concept through social media has fostered widespread awareness, identifying QQ as a growing threat to productivity and a potential catalyst for detrimental organizational culture [3]. Consequently, distortions and disruptions in employees’ work attitudes may arise, leading to dissatisfaction, disengagement, and contemplation of leaving their current positions [4].

Numerous studies have demonstrated that healthcare professionals (HPs) experience elevated levels of stress, anxiety, depression, and burnout [1, 5, 6]. Contributing factors include demanding workloads, extended working hours, exposure to high-risk environments, and concerns about infection [5, 7, 8]. In the post-pandemic period, HPs face additional challenges, including poor working conditions, income disparities, and work-life imbalances. These pressures are particularly evident among female HPs, who often struggle to balance family responsibilities with occupational demands. Furthermore, negative patient interactions and rising incidents of workplace violence [9, 10] have exacerbated emotional exhaustion and professional disengagement among HPs.

Within this context, the phenomenon of QQ—originally noted across various industries—has become increasingly relevant in healthcare settings [11]. The healthcare sector now faces growing instances of QQ, which threaten productivity and compromise patient care. Individuals engaging in QQ exhibit emotional and behavioral disengagement, which undermines critical aspects of care, including empathy, communication, and motivation. Given that successful patient outcomes rely on HPs’ dedication and commitment [11, 12], it becomes crucial to identify and address QQ to alleviate disengagement and enhance professional fulfillment.

In the contemporary work environment, QQ has emerged as a concept distinct from traditional burnout or fatigue. While burnout is characterized by emotional exhaustion and depersonalization, QQ reflects a different dimension—behavioral and attitudinal withdrawal. This subtle detachment may coexist with or follow burnout, emphasizing reduced motivation, loss of organizational commitment, and avoidance of professional interactions.

Recent comprehensive reviews have emphasized that QQ may not always represent maladaptive withdrawal but can, at early stages, function as a boundary-setting or coping mechanism to preserve mental health in demanding work environments [13]. However, when such detachment intensifies, it ceases to be protective and instead reflects emotional exhaustion and disengagement. Building on this distinction, the QQ phenomenon should be understood as a continuum ranging from adaptive self-protection to pathological disengagement.

The Maslach Burnout Inventory (MBI) and the Occupational Fatigue Exhaustion Recovery Scale (OFER) are well-established instruments widely used to assess burnout and occupational fatigue [14, 15]. The MBI evaluates emotional exhaustion, depersonalization, and reduced personal accomplishment, while the OFER measures acute and chronic fatigue as well as recovery between shifts. However, neither scale captures the behavioral disengagement and organizational withdrawal that characterize QQ. Therefore, a healthcare-specific measurement tool that integrates both emotional and behavioral indicators is necessary to comprehensively understand this emerging phenomenon.

Although several instruments measure work-related variables such as job satisfaction, burnout, turnover intention, and work engagement [16, 17], only one instrument has been developed to date to measure QQ among employees [18]. Nevertheless, its general structure and limited scope do not adequately reflect the distinctive working conditions of HPs, resulting in an incomplete assessment of their professional experiences. To address this gap, the present study aimed to develop a valid and reliable instrument to measure QQ specifically among HPs and to explore its underlying causes.

## Methods

### Research design

This study employed a descriptive survey design to identify and explore the phenomenon of QQ among HPs and to examine its underlying factors. The research was conducted in two stages: (1) the development and psychometric evaluation of the Quiet Quitting Scale for Health Professionals (QQS-HP), and (2) the implementation of the validated tool to determine the prevalence and predictors of QQ among HPs.

### Participants and data collection procedure

The study population consisted of HPs employed in public and private healthcare institutions across Türkiye. Participants were recruited voluntarily through online invitations distributed via institutional email lists, professional networks, and social media groups.

Inclusion criteria required participants to be actively employed in a healthcare setting and willing to participate voluntarily. Retired or administrative-only professionals were excluded. Data were collected between June and November 2024, and all participants provided electronic informed consent prior to participation.

The study was conducted in two phases. In the first phase, data were collected from a group of HPs (*n* = 242) for exploratory and confirmatory factor analyses to evaluate the structure and psychometric properties of the scale. In the second phase, the validated version of the scale was administered to a larger sample of HPs (*n* = 436) to assess the prevalence and associated factors of QQ. Ethical approval for the study was obtained from [insert institutional review board name and approval number].

### Scale development

The development of the QQS-HP followed established scale development and validation procedures [21]. An initial item pool was created based on an extensive literature review of studies addressing work disengagement, burnout, fatigue, and organizational withdrawal behaviors [1, 2, 7, 9, 11, 12, 18, 19].

A 25-item draft scale reflecting indicators of QQ and a 12-item list of possible causes were formulated. Content validity was assessed by eight experts in health management and behavioral sciences using Lawshe’s content validity ratio method [20]. Following expert feedback, minor linguistic and structural revisions were made.

A pilot test with 32 HPs was conducted to ensure item clarity and comprehensibility. No major revisions were required following the pilot phase. The pilot data were excluded from subsequent analyses.

### Data analysis plan

Data analysis included Exploratory Factor Analysis (EFA) to identify the factor structure of the QQS-HP, Confirmatory Factor Analysis (CFA) to validate the model, and internal consistency testing using Cronbach’s alpha coefficient. Test–retest reliability was evaluated using the Intraclass Correlation Coefficient (ICC) with a two-week interval.

These analyses were conducted to assess the construct validity, internal consistency, and temporal stability of the scale. Descriptive statistics, frequency distributions, and comparative analyses were performed using [insert software, e.g., SPSS version X and AMOS]. Detailed results of these psychometric analyses are presented in the Results section.

### Cut-off point determination

To facilitate the interpretation of QQS-HP scores, cut-off points were established based on the percentile distribution of total scores in the study sample. Specifically, the 25th, 50th, and 75th percentiles were calculated and used to define four severity categories: “no signs of QQ” (scores ≤ 40), “onset of QQ” (scores 41–48), “moderate QQ” (scores 49–56), and “severe QQ” (scores ≥ 57).

It is important to note that these cut-off points were derived from the present sample of HPs and reflect the unique occupational stressors and working conditions characteristic of healthcare settings. While these thresholds provide clinically meaningful reference values for this population, further validation studies are recommended to assess their applicability across different healthcare systems and occupational contexts.

## Results

### Scale validity and reliability analyses

Initially, Kaiser-Meyer-Olkin (KMO) and Bartlett’s tests were conducted to determine the suitability of the data for factor analysis. The results revealed a KMO value of 0.904, χ² value of 7715.354, and a p-value of 0.000, indicating that the data were appropriate for factor analysis [21].

Exploratory Factor Analysis (EFA) was conducted to ascertain the number of factors, explained variance ratio, and factor loadings of the items. To ensure a robust structure, item 21 with a factor loading below 0.35 was excluded. The remaining items were grouped under two factors. Aligned with the content of the items, Factor 1 was labeled “Employee Disengagement,” and Factor 2 as “Emotional Exhaustion and Depersonalization.” Subsequently, Confirmatory Factor Analysis (CFA) was performed to validate the factor structure derived from the EFA.

**Fig. 1:**
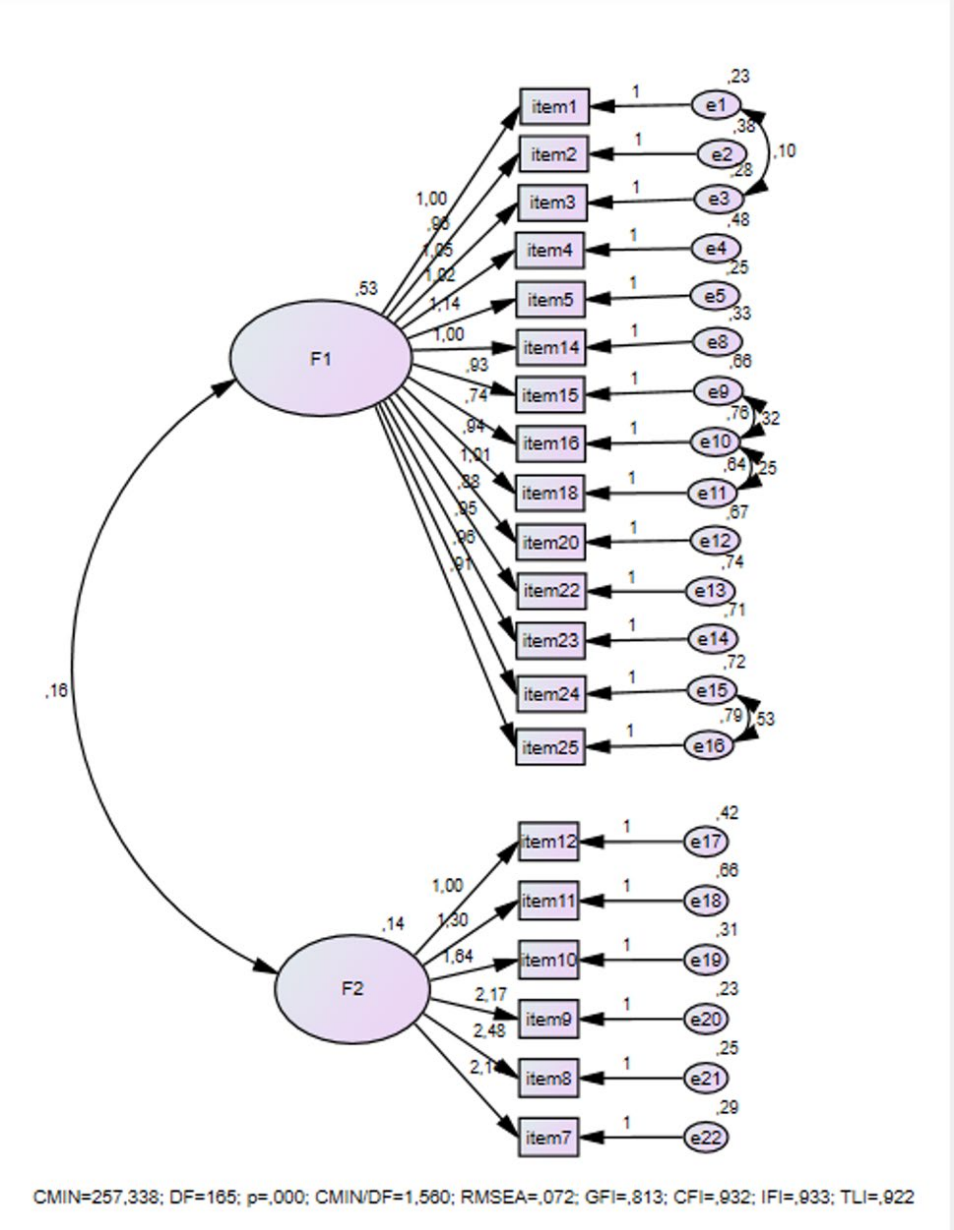
The Results of Confirmatory Factor Analysis. Note. CMIN = χ2: DF=Degrees of freedom: CFI= Comparative Fit Index: GFI=Goodness of Fit Index: TLI=Tucker-Lewis index: RMSEA=Root Mean Square Error of Approximation: IFI=Incremental Fit Index

The initial CFA included 24 items. During the analysis, items 6, 13, 17, and 19 demonstrated poor model fit and were consequently removed. A revised CFA was then conducted. Modification indices indicated high covariance between item 1 and item 3, item 15 and item 16, item 16 and item 18, as well as item 24 and item 25. Acceptable fit index values were achieved through the incorporation of error covariance. The findings from the CFA are presented in Fig. 1.

Following the CFA, the measurement model comprising 20 items and two subscales was validated. A subsequent EFA was conducted on the revised scale, and the outcomes are presented in Table 1.

**Table 1:** Descriptive and Psychometric Properties of Quiet Quitting Scale for Health Professionals (*n* = 242)

Items	Factor loading	M	SD	α^†^	*r*
Employee Disengagement					
Item 1. I do not want to come to the work.	0.949	2.73	0.88	0.937	0.74
Item 2. I want to leave the work early.	0.817	3.05	0.94	0.938	0.65
Item 3. I feel very uncomfortable when I come to work.	0.916	2.61	0.93	0.936	0.75
Item 4. I am unhappy at work.	0.704	3.03	1.02	0.938	0.67
Item 5. When my working hours are over, I don’t want to/stay for an extra 5 min at the workplace.	0.876	2.61	0.97	0.936	0.79
Item 14. I cannot work passionately and willingly.	0.712	2.41	0.94	0.936	0.76
Item 15. I feel that my relationships with my superiors are disconnected.	0.581	2.61	1.06	0.938	0.64
Item 16. I feel tense when my supervisors come to the clinic or when I meet them.	0.389	2.50	1.05	0.939	0.59
Item 18. I don’t want to participate in work meetings.	0.531	2.31	1.06	0.937	0.68
Item 20. I don’t want to answer calls/messages from work after working hours.	0.596	2.72	1.10	0.938	0.62
Item 22. I do not feel emotionally attached to my workplace.	0.588	2.50	1.08	0.939	0.57
Item 23. I only work to survive (to meet my basic needs).	0.557	2.88	1.09	0.938	0.64
Item 24. I wouldn’t want to do this job if I didn’t have to.	0.629	2.97	1.10	0.938	0.62
Item 25. If I had the chance, I would change my job.	0.673	3.06	1.11	0.939	0.56
Emotional Exhaustion and Depersonalization					
Item 7. When a patient/patient’s relative asks a question, I don’t want to/respond reluctantly.	0.791	2.00	0.96	0.938	0.61
Item 8. I don’t want to communicate verbally with patients/patient’s relatives unless it is necessary.	0.888	2.23	1.05	0.938	0.65
Item 9. I don’t want to/don’t ask “how are you” to patients/patient’s relatives during the day.	0.870	2.02	0.94	0.939	0.59
Item 10. Smiling at patients/patient’s relatives doesn’t come naturally to me/I don’t smile.	0.730	1.75	0.83	0.939	0.57
Item 11. Even if I know it would be helpful to the patient, I don’t do anything beyond my job description.	0.494	1.83	0.95	0.942	0.39
Item 12. I don’t communicate with my team members unless it is necessary.	0.353	1.81	0.75	0.940	0.53
Explained Variance Ratio	%50.746				

*α †* Cronbach’s alpha if item deleted, *r* Corrected item-total correlation, *M *Mean, *SD *Standard Deviation

Reliability analyses were performed using the test-retest method and Cronbach’s alpha coefficient. Test-retest reliability was assessed using the Intraclass Correlation Coefficient (ICC), yielding excellent results for the Employee Disengagement subscale (ICC = 0.997), the Emotional Exhaustion and Depersonalization subscale (ICC = 0.976), and the overall scale (ICC = 0.996). Cronbach’s alpha coefficients were 0.94 for the Employee Disengagement subscale, 0.87 for the Emotional Exhaustion and Depersonalization subscale, and 0.94 for the total QQS-HP.

### Scale scoring

The final 20-item scale and its subscales were structured as a 4-point Likert-type scale, with response options ranging from “never = 1 point,” “sometimes = 2 points,” “often = 3 points,” to “always = 4 points.” The scale contains no reverse-coded items.

In this study, cut-off points were established based on the percentile distribution of total QQS-HP scores. The 25th percentile corresponded to a score of 41, the 50th percentile to 49, and the 75th percentile to 57. These thresholds were used to define four severity categories, derived specifically from healthcare professionals and should be interpreted within comparable occupational contexts:

- 20 ≤ score ≤ 40: No signs of QQ
- 41 ≤ score ≤ 48: Onset of QQ
- 49 ≤ score ≤ 56: Moderate QQ
- Score ≥ 57: Severe QQ

### Prevalence and distribution of QQ

Table 2 presents the mean scores of HPs on the QQS-HP and its subscales. The mean score for the Employee Disengagement subscale was 45.78 ± 9.18, while the Emotional Exhaustion and Depersonalization subscale scored 13.55 ± 4.02. The overall mean score for the scale was 59.33 ± 11.83. Based on the established cut-off points, 23.9% of participants showed no signs of QQ, 24.8% exhibited onset of QQ, 22.0% experienced moderate QQ, and 29.4% reported severe QQ.

**Table 2:** The mean scores of health professionals on the quiet quitting scale for health professionals

	M ± SD	
Employee Disengagement	45.78 ± 9.18	
Emotional Exhaustion and Depersonalization	13.55 ± 4.02	
QQS-HP – Total Score	59.33 ± 11.83	
	n	%
QQS-HP		
No signs of QQ	104	23.9
Onset of QQ	108	24.8
Moderate QQ	96	22.0
Severe QQ	128	29.4

*QQ *Quiet Quitting, *QQS-HP *Quiet Quitting Scale for Health Professionals, *M *Mean, *SD *Standard Deviation

### Participant characteristics

Information on participants’ characteristics is shown in Table 3. The mean age of participants was 29.17 ± 6.14 years, the mean work experience was 6.79 ± 6.42 years, and the mean weekly working hours were 45.94 ± 8.68 h. Furthermore, 61.5% of participants were female, 53.2% held a bachelor’s degree, 55.0% were single, and 66.9% identified as nurses/midwives. Additionally, 75.2% of participants did not have children, and 60.6% worked in intensive care, emergency, or internal and surgical wards.

**Table 3:** Comparison of mean scores of the Quiet Quitting Scale for Health Professionals and its subscales according to socio-demographic and clinical characteristics of Health Professionals

Variables	Quiet Quitting Scale for Health Professionals		
	Employee Disengagement	Emotional Exhaustion and Depersonalization	Total
Age (29.17 ± 6.14 years)	*r = − *.124^**^ *p*=.009	*r* = −.137^**^ *p=*.004	*r *= −.143^**^ *p*=.003
Years of working (6.79 ± 6.42 years)	*r*= − .114 ^*^ *p*= .017	*r * = − .146 ^ **^ *p*= .002	*r* = − .138 ^**^ *p*= .004
Weekly working hours (45.94 ± 8.68)	*r=*.077 *p=*.107	*r = −*.019 *p=*.698	*r=*.054 *p=*.265
Gender			
Female (%61.5)	44.72 ± 8.69	12.88 ± 3.79	57.60 ± 11.18
Male (%38.5)	47.48 ± 9.68	14.61 ± 4.14	62.10 ± 12.34
*t=* *p=*	3.087 0.002	4.488 0.000	3.927 0.000
Education level			
Associate degree (%22)	46.57 ± 9.88^a^	14.87 ± 4.69^a^	61.44 ± 13.06^a^
Bachelor’s Degree (%53.2)	46.39 ± 9.37^a^	13.29 ± 4.00^a^	59.68 ± 12.02^a^
Graduate Degree (%24.8)	43.76 ± 7.80^b^	12.92 ± 3.05^b^	56.69 ± 9.71^b^
*F=* *p=*	3.513 0.031	7.182 0.001	4.388 0.013
Marital status			
Married (%45)	45.44 ± 9.80	14.20 ± 4.19	59.64 ± 12.87
Single (%55)	46.06 ± 8.65	13.01 ± 3.80	59.07 ± 10.93
*t=* *p=*	0.699 0.485	3.066 0.002	0.499 0.618
Having a child			
Yes (%24.8)	46.95 ± 10.48	14.70 ± 4.76	61.65 ± 14.16
No (%75.2)	43.38 ± 8.64	13.50 ± 3.75	56.88 ± 10.93
*t=* *p=*	2.180 0.031	0.405 0.001	1.496 0.001
Profession			
Nurse/Midwife (%66.9)	49.20 ± 9.10^a^	15.62 ± 5.00^a^	64.83 ± 12.25^a^
Medical doctor (%11)	48.12 ± 9.13^a^	14.16 ± 2.29^a^	62.28 ± 11.97^a^
Others (Technician, physiotherapist, nutritionist) (%22.1)	44.85 ± 9.24^b^	12.76 ± 3.61^b^	57.61 ± 11.69^b^
*F=* *p=*	8.887 0.000	20.573 0.000	14.301 0.000
Working clinic			
Intensive care (%19.3)	46.04 ± 10.42	13.47 ± 4.20	59.52 ± 13.37
Internal Medicine/Surgical clinics (%26.6)	46.15 ± 7.79	14.06 ± 3.68	60.22 ± 10.33
Emergency (%14.7)	46.26 ± 7.41	13.43 ± 2.16	59.70 ± 7.78
Others (%39.4)	45.22 ± 10.00	13.27 ± 4.63	58.50 ± 13.19
*F=* *p=*	0.355 0.786	0.930 0.426	0.526 0.665

F = One-way ANOVA: t=Independent Samples t Test

a-b=There is no statistically significant difference between values with the same letter

*Correlation is significant at the 0.05 level (2-tailed)

**Correlation is significant at the 0.01 level (2-tailed)

### Comparative analysis of QQS-HP scores

The comparative analysis of mean scores on the QQS-HP and its subscales according to socio-demographic and clinical characteristics of participating HPs is presented in Table 3. No statistically significant differences were observed in mean scores concerning weekly working hours or work setting (*p* >.05).

As age and years of work experience decreased, the mean scores for both the Employee Disengagement and Emotional Exhaustion and Depersonalization subscales, as well as the total QQS-HP score, increased. Furthermore, higher mean scores on all subscales and the overall QQS-HP were observed among male participants, those with children, and nurses, midwives, and medical doctors (*p* <.05).

Significant differences were observed in mean scores of both subscales and the overall QQS-HP based on educational background, with lower scores among individuals with postgraduate or medical education compared to those with undergraduate education (*p* <.05).

A statistically significant positive correlation was found between the subscales of the QQS-HP. Employee Disengagement was moderately correlated with Emotional Exhaustion and Depersonalization (*r* =.537, *p* <.001). Furthermore, both subscales demonstrated strong positive correlations with the total QQS-HP score (Employee Disengagement: *r* =.958, *p* <.001; Emotional Exhaustion and Depersonalization: *r* =.756, *p* <.001).

The reasons for QQ among HPs who experienced severe QQ (*n* = 128; 29.4%) are presented in Table 4. The analysis reveals that the factors contributing to QQ are overwhelmingly related to systemic and managerial stressors, with four factors being cited by over 90% of participants.

**Table 4:** Frequency table of the causes of quiet quiting syndrome (*n* = 128)

Reasons	*n*	%
Unfavorable working conditions	116	90.6
Increased workload	120	93.8
Low wages	116	90.6
Ministry policies	88	68.8
Communication problems	88	68.8
Physical and verbal violence from the patient/patient relatives	88	68.8
Psychological violence from team members	60	46.9
Psychological violence from managers	116	90.6
Managers do not value employees’ opinions	108	84.4
Disturbed work-life balance (too much time at work and not enough time for social life)	112	87.5
Insufficient use/restriction of annual leave	100	78.1
Lack of appreciation	104	81.3

Those who answered “always” to any of the Quiet Quitting Scale for Health Professionals items were asked to indicate the reason(s) for this situation. Participants were able to tick more than one option

The most frequently cited reason was increased workload (93.8%), followed by psychological violence from managers (90.6%), unfavorable working conditions (90.6%), and low wages (90.6%). These four factors emerged as the primary drivers of severe QQ, each affecting more than nine out of ten HPs in this group.

Beyond these predominant issues, several additional factors were identified by a substantial proportion of participants: disturbed work-life balance (87.5%) and managers not valuing employees’ opinions (84.4%) were cited by more than four-fifths of the group. Furthermore, ministry policies (68.8%), communication problems (68.8%), and physical and verbal violence from patients/patient relatives (68.8%) were each reported by approximately two-thirds of participants. Psychological violence from team members was cited by 46.9% of HPs experiencing severe QQ. The prevalence of multiple organizational, interpersonal, and systemic factors demonstrates that the causes of severe QQ are pervasive and multidimensional within this group.

## Discussion

### Development of QQS-HP

PubMed, Medline, Scopus, CINAHL, Cochrane, and ISI Web of Knowledge databases were systematically reviewed to construct the initial item pool of the QQS-HP. The reliability and validity of the scale were rigorously tested through psychometric analyses. Exploratory and confirmatory factor analyses revealed a robust two-factor structure consisting of 20 items, with strong internal consistency (Cronbach’s α = 0.94) and excellent test–retest reliability (ICC = 0.996), confirming the stability of the instrument over time.

The two subscales of the QQS-HP were identified as Employee Disengagement (14 items) and Emotional Exhaustion and Depersonalization (6 items). The first subscale measures behavioral and cognitive withdrawal—such as avoidance of communication, reluctance to participate in meetings, and disengagement from organizational goals—whereas the second captures the emotional and interpersonal manifestations of exhaustion, including loss of empathy and depersonalized attitudes toward patients and colleagues. As Di Monte et al. [22] highlighted, such emotional depletion impairs HPs’ capacity for empathy and interpersonal connection, reflecting the core psychological mechanisms measured by this scale.

Given that QQ encompasses emotional, cognitive, and behavioral withdrawal, it can be more accurately conceptualized as a syndrome rather than a single psychological state. A syndrome represents a constellation of interconnected feelings, thoughts, and behaviors that collectively characterize a specific phenomenon [23]. Thus, in this study, the construct is referred to as Quiet Quitting Syndrome (QQS) to emphasize its multidimensional and systemic nature. This conceptual framing aligns with contemporary literature suggesting that QQ unfolds along a continuum—from adaptive coping or boundary-setting to maladaptive emotional disengagement and depersonalization [13]. In its early, mild stages, QQS may serve as a self-protective mechanism that enables HPs to preserve mental health and prevent burnout. However, as disengagement intensifies, it ceases to be protective and becomes a maladaptive form of emotional exhaustion and detachment. The QQS-HP was designed to detect and differentiate these stages, offering researchers and practitioners a means to identify early signs of withdrawal before they evolve into full disengagement or burnout.

### Comparison with existing scales

The QQS-HP extends beyond existing instruments such as the MBI and the OFER by capturing behavioral and attitudinal aspects that traditional burnout measures overlook.

While the MBI assesses emotional exhaustion, depersonalization, and reduced personal accomplishment, it focuses primarily on the psychological manifestations of burnout and fails to account for the behavioral disengagement that defines QQS. Similarly, the OFER measures acute and chronic fatigue and post-shift recovery, but it does not address interpersonal detachment or organizational withdrawal.

In contrast, the QQS-HP integrates both emotional and behavioral indicators, encompassing dimensions such as avoidance of teamwork, communication withdrawal, reluctance to engage in meetings, and loss of professional motivation. Furthermore, the inclusion of empirically derived cut-off points allows classification of QQS severity (no signs, onset, moderate, severe), providing a diagnostic framework for monitoring disengagement progression.

Rather than replacing existing measures, the QQS-HP complements them, offering a healthcare-specific, context-sensitive, and multidimensional approach to evaluating QQ. This makes it particularly valuable for identifying early behavioral and emotional signals of disengagement in healthcare environments, where employee commitment directly influences patient safety, satisfaction, and overall quality of care.

### Identification of QQS and its causes

The mean score obtained from the QQS-HP among HPs was 59.33 ± 11.83, indicating that most participants experienced moderate to severe levels of QQS. Only 23.9% showed no signs of the syndrome, while 76.1% reported varying degrees—ranging from onset (24.8%) to moderate (22.0%) and severe (29.4%) QQS. These findings reveal that QQS is a critical and prevalent issue within healthcare, potentially compromising the quality of patient care [24].

When compared with the study by Galanis et al. [18], which reported that 60.9% of nurses experienced QQ, the QQS-HP provides a more nuanced assessment. The earlier instrument, not tailored for healthcare contexts, offered only a binary outcome (presence or absence of QQ) and lacked evaluation of severity. In contrast, the QQS-HP identifies distinct levels of disengagement, making it more suitable for clinical and organizational applications.

In our study, the mean score for the Employee Disengagement subscale among HPs was 45.78 ± 9.18, indicating a substantial level of employee disengagement within the sample. Employee disengagement is a critical concept that adversely impacts the performance of individuals in the workforce and diminishes the quality of healthcare services. For HPs, who are dedicated to improving patients’ health, this represents an even more significant concern. Aspects such as patient safety, frequency of medical errors, healthcare costs, and patient satisfaction are influenced by employee commitment [25–27]. When examining the relationship between the QQS-HP and its subscales, a strong correlation was observed between increasing employee disengagement and the phenomenon of QQS. Therefore, it is imperative to closely monitor employee disengagement, diagnose it early, and prevent QQS through appropriate interventions.

In their recent opinion article exploring the impact of QQ on healthcare quality, Boy and Sürmeli [12] suggested that emotional exhaustion and depersonalization could serve as precursors to QQS. The results of the current study support these claims, showing a robust association between increased emotional exhaustion and depersonalization and QQS. Our research revealed a mean score of 13.55 ± 4.02 for the Emotional Exhaustion and Depersonalization subscale, indicating a moderate to severe level of these phenomena among HPs. Key aspects such as neglecting to respond to patients, disregarding emotional needs, and avoiding communication are fundamental components of healthcare quality. The deterioration of these elements contributes to a decline in healthcare service quality and negatively impacts patient satisfaction [28, 29]. In addition, desensitization may lead to overlooking the early detection of critical complications, potentially reducing patients’ quality of life, increasing morbidity and mortality, and escalating healthcare costs. It is therefore imperative to closely monitor emotional exhaustion and depersonalization and to take proactive measures to reduce the risk of QQS through early diagnosis of symptoms.

The study found a consistent trend: as age and years of work experience decreased, there was a corresponding increase in mean scores on the QQS-HP and all its subscales. It is widely acknowledged in the global literature that generational differences, particularly among Generation Z, may be a contributing factor to the prevalence of QQS, with Generation Z being identified as more susceptible to this phenomenon [19, 30–32]. In support of these claims, the results of our study highlight a greater susceptibility to QQS among individuals with younger age and fewer years of work experience. While the older generation in the workforce emphasizes obedience and a comfortable lifestyle, the new generation emphasizes achieving work-life balance and expectations of recognition [33]. Gallup [34] reports that 73% of American Gen Z employees have left their jobs because their workplace expectations were not met, and Deloitte found that 61% of Gen Z employees plan to leave their jobs in the next two years. Given the increasing proportion of Gen Z in the healthcare workforce [12], it is imperative to thoroughly assess and address the potential for this demographic to experience QQS to ensure the delivery of quality healthcare services.

The results indicate that male HPs have higher mean scores on the QQS-HP and all its subscales compared to their female counterparts. First, it is essential to contextualize this finding within the broader structural realities of the sector. Given that healthcare is a disproportionately female sector, the established challenges—such as poor working conditions, income disparities, and work-life imbalances—are generally reported to be more evident and acutely felt among female HPs globally [35, 36]. Therefore, our finding that male HPs reported higher QQS-HP scores is a particularly noteworthy observation that deviates from this general expectation. The variance in gender-related outcomes may be due to cross-cultural differences or the specific nature of the QQS-HP instrument. For example, in Türkiye, despite the increasing role of women in the labor market, a prevailing norm still holds that women are primarily responsible for domestic work and childcare. These norms, while potentially increasing the total workload on female HPs, may also contribute to women’s higher psychological resilience compared to men [37], which might be a factor preventing higher QQS-HP scores. Furthermore, it is worth noting that nursing is generally perceived as a career preferred by women in Turkish society [38–40]. Our finding that male HPs scored higher on the QQS-HP could thus imply a greater professional or psychological risk for male nurses in this context. This risk may manifest as lower professional satisfaction, which could lead to the reported higher QQS-HP scores. In the international literature, there is evidence to support the likelihood of male nurses experiencing higher levels of burnout [41, 42], which aligns with our finding of higher QQS-HP scores among males. All these findings highlight the need for further research into how gender differences, sectoral structural issues, and societal norms may influence the experiences of HPs with QQS.

Those with postgraduate or medical education were found to have lower mean scores on the QQS-HP and all subscales compared to other graduates. This finding suggests that as the level of education increases, the incidence of QQS tends to decrease. The study by Abun et al. [43] showed that employees with higher levels of education tend to have higher levels of self-efficacy, which has a positive effect on employee engagement. Furthermore, a meta-analysis by Alsaiari et al. [44] showed that employees with higher educational levels have lower job turnover rates. Higher levels of education tend to increase professional competence and contribute to improving the quality of care. In this context, it can be said that HPs with higher levels of education have higher levels of employee engagement, which leads to lower risks of emotional exhaustion and depersonalization. In contrast to studies suggesting that task–skill mismatch may increase the risk of QQ [13], our findings showed that HPs with postgraduate or medical education exhibited lower levels of QQS. This discrepancy can be attributed to contextual and structural characteristics of the healthcare system. In clinical environments, higher education typically grants professionals greater autonomy, decision-making authority, and alignment between their competencies and job roles, which mitigate the perception of skill underutilization. Consequently, the task–skill match within highly trained healthcare roles likely acts as a protective factor against disengagement.

Conversely, in occupational contexts where individuals’ educational qualifications exceed job requirements or where professional autonomy is constrained, task–skill mismatch may emerge as a risk factor for QQS, as highlighted by Bernuzzi et al. [13]. Therefore, our results suggest that the relationship between education level and QQ is context-dependent, varying according to whether higher education enhances or frustrates role congruence and professional fulfillment.

Our research has shown that those who are married and have children tend to experience higher levels of emotional exhaustion and depersonalization. This tendency is thought to be due to efforts to maintain work-life balance. HPs may experience energy constraints at work to devote more time to their families and children [45, 46]. This situation may manifest itself in more detached behavior at work. In addition, those with families and children may experience a higher incidence of employee disengagement and be more susceptible to QQS. This could be attributed to an imbalance between work and life. Indeed, this scenario is presented as a possibility in the current literature [12, 19, 34].

In this study, it has been identified that nurses, midwives, and medical doctors experience higher levels of employee disengagement, emotional exhaustion, depersonalization, and QQS compared to other HPs. We attribute this situation to the obligation of nurses, midwives, and medical doctors to provide services 24/7. Additionally, constant communication with patients and the necessity of providing one-on-one care may have emotionally drained them and led to the development of QQS. These findings indicate a heightened risk of emotional exhaustion and depersonalization among HPs, particularly nurses, midwives, and doctors in the healthcare industry. Notably, there is a lack of studies in the literature specifically addressing the levels of QQS among HPs in relation to their occupations.

The results of the present study also support the multidimensional perspective proposed by Bernuzzi et al. [13], who emphasized that QQ should be conceptualized along a continuum—from adaptive coping to maladaptive disengagement. At mild levels, QQS may serve as a self-protective mechanism that enables HPs to maintain psychological boundaries and prevent burnout in high-stress environments. However, when this behavioral withdrawal becomes persistent and intensified, it loses its protective value and transforms into emotional exhaustion and depersonalization. In this sense, the moderate and severe levels of QQS observed in our study are indicative not of adaptive self-regulation but of a maladaptive phase in which HPs have already reached psychological depletion. Recognizing this continuum is essential for developing early intervention strategies that can detect and manage mild, adaptive forms of QQS before they evolve into full disengagement or burnout.

Research examining the factors that lead HPs to resign in different countries shows that factors affecting employee satisfaction, such as job satisfaction and quality of life, are prominent [47–50]. However, there is a notable lack of research that specifically addresses the reasons that lead both HPs and staff in different institutions to QQS. The only research study on this topic is by Galanis et al. [24]. This study assessed the impact of job burnout on QQ and the mediating role of job satisfaction. The results indicated that job burnout increased QQ, while job satisfaction decreased it. Factors influencing job burnout and job satisfaction include heavy workload, dissatisfaction with pay, work-life imbalance, unprofessional management approaches, and unfavorable working conditions [51, 52]. Similarly, the results of our study show that among the factors contributing to QQS among HPs, increased workload, unfavorable working conditions, and psychological violence from managers were the most prominent. These factors reduce employee engagement and lead to employee burnout. Reduced employee engagement and burnout critically affect employee performance, productivity, organizational outcomes, and quality of care.

### Theoretical and practical implications

The findings of this study contribute to the growing body of literature on work disengagement by framing QQ as a multidimensional construct that encompasses emotional, cognitive, and behavioral components. By conceptualizing the phenomenon as QQS, this study advances the theoretical understanding of disengagement as a continuum ranging from adaptive self-protection to maladaptive withdrawal. This conceptualization provides a framework for future research to explore the transition points between these phases and to investigate the role of contextual moderators such as organizational culture, leadership style, and workload.

From a practical perspective, the QQS-HP offers healthcare administrators, policymakers, and researchers a context-sensitive tool to identify early signs of disengagement among HPs. Routine monitoring of QQS levels may guide the implementation of preventive interventions such as promoting supportive leadership, optimizing workload distribution, and enhancing job autonomy. Furthermore, the scale can serve as a diagnostic tool in both organizational audits and research studies aiming to evaluate the impact of workplace interventions on employee engagement, burnout prevention, and quality of care outcomes.

### Limitations

Despite its strengths, this study has several limitations. First, the present study focused on establishing the internal structure, reliability, and stability of the QQS-HP. Due to the scope of data collection, discriminant or convergent validity analyses with external constructs (such as burnout or fatigue) were not performed. The conceptual framework of the QQS-HP distinguishes QQ from burnout by emphasizing behavioral and attitudinal withdrawal beyond emotional exhaustion and depersonalization. Future studies should further examine criterion and predictive validity by comparing QQS-HP with established instruments such as the MBI and the OFER to more precisely differentiate QQS from related constructs. Second, the cross-sectional design restricts the ability to infer causal relationships between QQS and its associated factors. Longitudinal studies are recommended to examine the temporal dynamics of QQS and its potential evolution from adaptive to maladaptive disengagement. Third, the data were collected through self-report measures, which may be subject to response bias and social desirability effects. Triangulating these findings with qualitative interviews or supervisor evaluations would strengthen future research. Fourth, the study sample was limited to HPs in Türkiye, which may restrict the generalizability of the findings to other cultural or occupational contexts. Validation studies across diverse healthcare systems and professional groups are necessary to establish the broader applicability of the QQS-HP.

## Conclusions

The QQS-HP is a valid and reliable measurement tool designed to identify both the presence and severity of QQS among HPs. The findings reveal that while mild levels of QQS may reflect adaptive coping or boundary-setting mechanisms that help HPs preserve psychological well-being in demanding work environments, moderate and severe levels indicate maladaptive disengagement characterized by emotional exhaustion and depersonalization.

This distinction underscores the need to interpret QQS as a continuum rather than a purely negative state. Early identification of mild, adaptive forms of disengagement may present an opportunity for preventive interventions that protect HPs from progressing to burnout or complete withdrawal. However, persistent and intensified QQS behaviors pose a significant risk to healthcare quality, as HPs may limit their engagement to only basic responsibilities, neglecting the emotional support, empathy, and communication that are essential for patient-centered care.

Considering that HPs’ engagement directly affects patient safety, satisfaction, and treatment outcomes, healthcare institutions should monitor early signs of disengagement and implement organizational strategies to promote well-being, fair workload distribution, and supportive leadership. Addressing QQS through early detection and targeted interventions will not only enhance employee satisfaction but also sustain the quality and continuity of healthcare services.

## Data Availability

The dataset used and analyzed during the current study is available from the corresponding author on reasonable request.

## Abbreviations

QQ: Quiet Quitting
QQS: Quiet Quitting Syndrome
HPs: Health Professionals
QQS-HP: Quiet Quitting Scale for Health Professionals
KMO: Kaiser-Meyer-Olkin
EFA: Exploratory Factor Analysis
CFA: Confirmatory Factor Analysis
ICC: Intraclass Correlation Coefficient
